# Early awake prone position combined with high-flow nasal oxygen therapy in severe COVID-19: a case series

**DOI:** 10.1186/s13054-020-02991-7

**Published:** 2020-05-24

**Authors:** Qiancheng Xu, Tao Wang, Xuemei Qin, Yanli Jie, Lei Zha, Weihua Lu

**Affiliations:** 1 Department of Critical Care Medicine, The First Affiliated Hospital of Wannan Medical College (Yijishan Hospital of Wannan Medical College), No.2, West road of Zheshan, Jinghu District, Wuhu, 241000 Anhui China; 2 Department of Critical Care Medicine, The Second People’s Hospital of Wuhu, No 265, Jiuhua Road, Jinghu District, Wuhu, 241000 Anhui China; 3 Institute of Infection and Global Health, University of Liverpool, Liverpool, L69 7BE UK

**Keywords:** COVID-19, High-flow nasal cannula, Prone positioning, SARS-CoV-2

Coronavirus disease 2019 (COVID-19) has rapidly spread around the world in the past 3 months and has now become a global public health crisis. The mortality of COVID-19 in some European cities exceeds 11%, and the fatality rate is up to 61.5% in critical patients, especially in mechanically ventilated patients [1]. Once mild to moderate patients progress to critical illness, the incidence of septic shock, intubation, and myocardial injury increases significantly. Mechanical ventilation patients need more sedative, analgesic, and neuromuscular blocker drugs, which will affect the patient’s hemodynamic status and airway expectoration ability [2]. Previous studies have confirmed that high-flow nasal cannula (HFNC) can reduce the endotracheal intubation rate and mortality in patients with respiratory failure [3]. However, this therapy of COVID-19 cannot improve the pathophysiology of ventilation-perfusion defects and atelectasis, which can be proved by autopsies, i.e., small airways are blocked by mucus plugs [4]. Awake prone position could improve the mismatch of ventilation-perfusion and open the atelectatic lungs by adequate sputum drainage. So far, the role of early awake prone position (PP) combined with HFNC therapy in the treatment of severe COVID-19 has not been reported. So, we conducted a retrospective observation study in three hospitals in Wuhu and Maanshan cities in Anhui Province.

From January 1 to April 2, 2020, 79 patients with coronavirus infection were screened. Ten was severe and all of them received early awake PP combined with HFNC treatment (Table 1). COVID-19 was diagnosed using sputum or throat swab determined by real-time reverse transcription polymerase chain reaction (RT-PCR) assay. The severity of disease was graded according to the Guidelines for the Diagnosis and Treatment of Novel Coronavirus (2019-nCoV) Infection by the National Health Commission (trial version 5). The target time of prone positioning is more than 16 h per day and can be appropriately shortened according to the patient’s tolerance. Target SpO_2_ was more than 90% of adult non-pregnant patients (Fig. 1a).

**Table 1:** Clinical characteristics and outcomes of COVID patients treated by prone position combine with HFNC

Case no.	Gender	Age (years)	Comorbidity	Imagine features	Time from illness onset to hospitalization	Baseline PF (onset of HFNC) (mmHg)	WBC (× 10^9^/L)	L (× 10^9^/L)	PCT (ng/ml)	CRP (mg/L)	D-dimer (mg/L)	Intubation	Length of stay (days)	Outcome
1	Male	54		Bilateral lobes, GGO and consolidation	10	156.8	5.2	0.8	0.1	111.2	2.4	No	18	Survivor
2	Female	56	DM	Bilateral lobes, GGO	5	169.2	5.4	1.0	0.3	8.2	1.0	No	19	Survivor
3	Male	47	DM, HBP	Bilateral pulmonary infiltration	10	123.6	9.6	1.0	0.2	84.0	0.2	No	11	Survivor
4	Female	65		Bilateral lobes, GGO	6	117.7	6.6	0.4	0.2	97.3	3.0	No	11	Survivor
5	Female	51	HBP	Bilateral pulmonary infiltration	10	205.8	6.2	0.6	0.1	110.2	0.6	No	11	Survivor
6	Male	43		Bilateral lobes, GGO and consolidation	6	188.8	2.2	0.8	< 0.1	27.7	1.2	No	15	Survivor
7	Female	48	HBP	Bilateral lobes, GGO and consolidation	9	89.1	4.7	0.5	< 0.1	47.4	0.9	No	30	Survivor
8	Female	51		Bilateral lobes, GGO and consolidation	5	155.5	9.5	2.0	< 0.1	68.3	0.4	No	22	Survivor
9	Male	56	HBP	Bilateral lobes, GGO and consolidation	6	227.8	3.6	1.1	< 0.1	15.3	2.3	No	19	Survivor
10	Male	31		Bilateral lobes, GGO and consolidation	1	134.7	4.2	1.4	0.2	9.5	1.9	No	21	Survivor

**Fig. 1:**
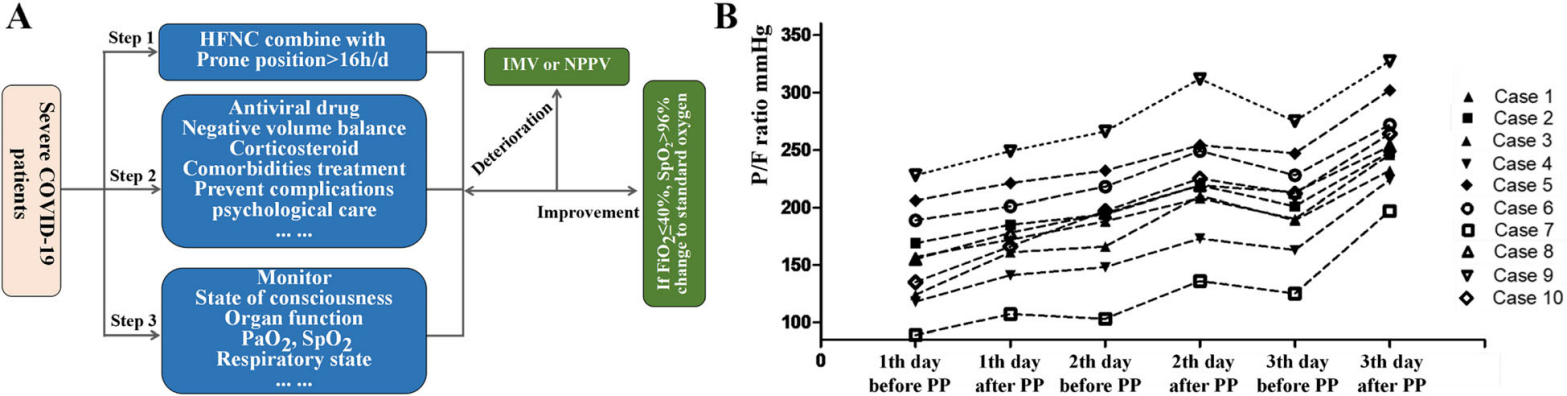
**a** The protocol treatment of severe COVID-19 patients. **b** The change of PaO_2_/FiO_2_ in the first 3 days between onset of HFNC and 4–6 h after PP therapy in severe COVID-19 patients

All the severe patients, with PF < 300 mmHg, developed mild respiratory alkalosis and no alkalemia at the beginning of HFNC treatment. After PP, compared with the baseline, the median PaCO_2_ increases slightly [32.3 (29.3–34.0) vs. 29.7 (28.0–32.0), *p* < 0.001]. The median PaO_2_/FiO2 (PF) was elevated significantly after PP (Fig. 1b). None of the patients progressed to critical condition or needed endotracheal intubation.

When the condition of patients with severe coronavirus deteriorated rapidly and the mortality rate was unacceptably high [1], we then put forward a concept “reduce the proportion of severe COVID-19 conversion to critical illness,” based on the experience summarized during the treatment of COVID-19 in Anhui, China. Early awake PP combined with HFNC therapy was one of the most important strategies to avoid intubation and reduce the requirement for medical staff. A previous study reported that early application of PP with HFNC therapy, especially in patients with moderate ARDS, may help avoid intubation but not in the several ARDS group [5]. However, in non-infected severe ARDS patients with a PF < 100 mmHg, 5 of 6 patients avoided intubation. In our research, the lowest P/F ratio was 89 mmHg, and the patient was successfully discharged without invasive mechanical ventilation. The main reason for the patients’ intolerance of PP is discomfort, anxiety [6], and the inability to change position. Our strategy was psychological care and a slight change of position every 2 h.

Compared to non-invasive ventilation (NIV), patients felt more comfortable when using HFNC therapy, and the demand for medical staff was reduced. Awake PP combined with HFNC therapy could be used safely and effectively in severe COVID-19 patients, and it may reduce the conversion to critical illness and the need for tracheal intubation.

## Data Availability

Data are available on request.

## Abbreviations

COVID-19: Coronavirus disease 2019
HFNC: High-flow nasal cannula
PP: Prone position
PF: PaO_2/_FiO_2_
NIV: Non-invasive ventilation

## References

[1] Yang X, Yu Y, Xu J, Shu H, Xia J, Liu H, Wu Y, Zhang L, Yu Z, Fang M, et al. Clinical course and outcomes of critically ill patients with SARS-CoV-2 pneumonia in Wuhan, China: a single-centered, retrospective, observational study. Lancet Respir Med. 2020;8(5):475–81. 10.1016/S2213-2600(20)30079-5 PMC7102538 32105632

[2] Bellani G, Laffey JG, Pham T, Fan E, Brochard L, Esteban A, Gattinoni L, van Haren F, Larsson A, McAuley DF (2016). Epidemiology, patterns of care, and mortality for patients with acute respiratory distress syndrome in intensive care units in 50 countries. JAMA.

[3] Thille AW, Muller G, Gacouin A, Coudroy R, Decavele M, Sonneville R, Beloncle F, Girault C, Dangers L, Lautrette A (2019). Effect of postextubation high-flow nasal oxygen with noninvasive ventilation vs high-flow nasal oxygen alone on reintubation among patients at high risk of extubation failure: a randomized clinical trial. JAMA.

[4] Liu Q, Wang RS, Qu GQ, Wang YY, Liu P, Zhu YZ, Fei G, Ren L, Zhou YW, Liu L (2020). Gross examination report of a COVID-19 death autopsy. Fa Yi Xue Za Zhi.

[5] Ding L, Wang L, Ma W, He H (2020). Efficacy and safety of early prone positioning combined with HFNC or NIV in moderate to severe ARDS: a multi-center prospective cohort study. Crit Care.

[6] DiSilvio B, Young M, Gordon A, Malik K, Singh A, Cheema T (2019). Complications and outcomes of acute respiratory distress syndrome. Crit Care Nurs Q.

